# Effect of virtual reality on pain in oncology patients: A systematic review

**DOI:** 10.1097/MD.0000000000043487

**Published:** 2025-08-08

**Authors:** Ayşin Tepe, Elif Dönmez, Tülay Ortabağ

**Affiliations:** a Marmara University Training and Research Hospital, Clinical Nurse, Istanbul, Turkey; b Department of Oncology Nursing, University of Health Sciences Hamidiye Faculty of Nursing, Istanbul, Turkey; c Department of Nursing, Istanbul Topkapi University Faculty of Health Sciences, Istanbul, Turkey.

**Keywords:** cancer, oncology, pain, pain management, virtual reality

## Abstract

**Background:**

This study aims to evaluate the effectiveness of virtual reality (VR) applications in managing pain among cancer patients. Pain is a prevalent symptom in oncology and significantly impacts patients’ quality of life. VR, as a non-pharmacological intervention, distracts attention and provides immersive environments, making it a promising approach to alleviate pain. This systematic review synthesizes evidence from studies conducted between 2014 and 2024 to assess VR’s impact on pain management and its implications for clinical practice.

**Methods:**

A systematic literature review was conducted following Preferred Reporting Items for Systematic Reviews and Meta-Analyses guidelines. The PubMed, Web of Science, and Cochrane databases were searched via specific Medical Subject Headings terms. Studies were included if they were experimental or quasi-experimental, published in English, and accessible in full text. The population was composed of cancer patients, and the intervention was VR-based. Data extraction and quality assessment were performed independently by 2 researchers using Joanna Briggs Institute tools. From an initial pool of 497 studies, 22 met the inclusion criteria, including 10 randomized controlled trials and 12 quasi-experimental studies.

**Results:**

The included studies included 925 participants aged 6 to 85 years, representing various cancer types, such as breast, colorectal, and hematological malignancies. VR interventions were associated with significant pain reduction in 13 studies, while 6 studies reported no meaningful differences, and 1 study noted a slight adverse effect. The participants frequently reported enhanced satisfaction and emotional well-being, describing the VR experience as relaxing and distracting. Despite mild side effects such as nausea or dizziness, VR was generally well tolerated.

**Conclusion:**

VR has considerable potential as a complementary method for pain management in oncology. While it shows promise in improving patient experiences and reducing pain, further robust, large-scale studies are needed to validate its clinical effectiveness and optimize its use. These findings encourage healthcare professionals to explore VR as a holistic tool in cancer care.

## 1. Introduction

Cancer is a significant health issue both globally and in our country. The World Health Organization reported that in 2022, 20 million new cancer cases and 9.7 million cancer-related deaths occurred.^[1]^ In Turkey, cancer-related deaths rank second among all causes of death.^[2]^ with the development of technology and the increase in treatment options, the survival rate for cancer patients has increased, making symptom management even more critical in this process. Cancer patients face various physical and psychosocial symptoms due to the disease and its progression. One of the most common symptoms is pain.^[3–5]^

Among cancer patients, 20% to 30% experience pain, 60% to 100% of patients in advanced stages, 90% of terminal-stage patients, and 20% to 50% of cancer survivors experience pain at varying levels.^[4–7]^ Pain management in cancer patients includes pharmacological, surgical, and non-pharmacological methods. Among non-pharmacological methods, virtual reality (VR), skin stimulation, massage, hot–cold applications, music therapy, imagery, and distraction techniques are some of the applications used in pain management.^[8,9]^ The use of non-pharmacological methods in cancer patients is widely accepted as a standard for holistic care.^[8–10]^

Recent studies have indicated that VR is used among non-pharmacological methods for pain management.^[11–14]^ VR technology was first introduced in 1989 by Jaron Lanier.^[15–17]^ In oncology, Schneider conducted studies on children with leukemia and Hodgkin lymphoma.^[18]^ VR refers to a technology in which users interact with a virtual environment and perceive themselves as physically existing within that environment. The experience sends various stimuli to the brain by influencing the cognitive process of attention. The mechanism of action involves shifting the individual’s focus away from unpleasant pain sensations to the virtual environment. VR experiences can be either passive or actively engaged through hand movements.^[19]^ Studies on VR have reported that participants experience high levels of satisfaction and enjoyment.^[20,21]^ Healthcare professionals also exhibit a positive approach toward using VR.^[22,23]^ The application of VR has become more common in healthcare due to its reduced cost, ease of use, portability, and the fact that it does not require certification or complex procedures.^[15,18,23]^ In the literature, the effects of VR on pain have been investigated in various age groups, including pediatric oncology patients, individuals with breast cancer, those undergoing chemotherapy, and patients with solid tumors.^[19,24–26,27]^

Meta-analyses have demonstrated that VR-based interventions significantly reduce pain intensity in cancer patients compared to conventional rehabilitation methods. Particularly during painful procedures and treatment-related stress, VR applications have been shown to alleviate pain perception through their immersive and distracting effects.^[26]^ In addition, VR has been associated with supplementary clinical benefits such as reducing the need for medication, enhancing treatment adherence, and shortening hospital stays.^[28]^ While existing studies primarily address physical and psychological symptoms in general or focus on specific patient groups or age ranges, there remains a need for a more targeted perspective on pain-of the most prevalent and debilitating symptoms in cancer patients. Pain significantly impacts quality of life, and therefore, conducting a comprehensive review that encompasses all cancer patients, rather than focusing solely on specific subpopulations, is of critical importance. Developing a focused approach to pain management through VR not only has the potential to improve patients’ quality of life but also to support healthcare professionals in clinical decision-making processes.^[24,26,28]^

The purpose of this review is to determine the effects of interventions using VR on pain symptoms in cancer patients. This systematic review aims to provide guidance for oncology nurses on how to incorporate VR applications into their clinical practice and serve as a resource for non-pharmacological methods to alleviate pain.

The main research questions addressed in this study are as follows:

- To what extent are VR applications effective in pain management in cancer patients, and in which situations are they ineffective?
- Which VR content has been used for pain management in cancer patients?
- What is the relationship between patient satisfaction and the effectiveness of pain management in the use of VR applications?

## 2. Method

### 2.1. Research design

**Figure 1.**
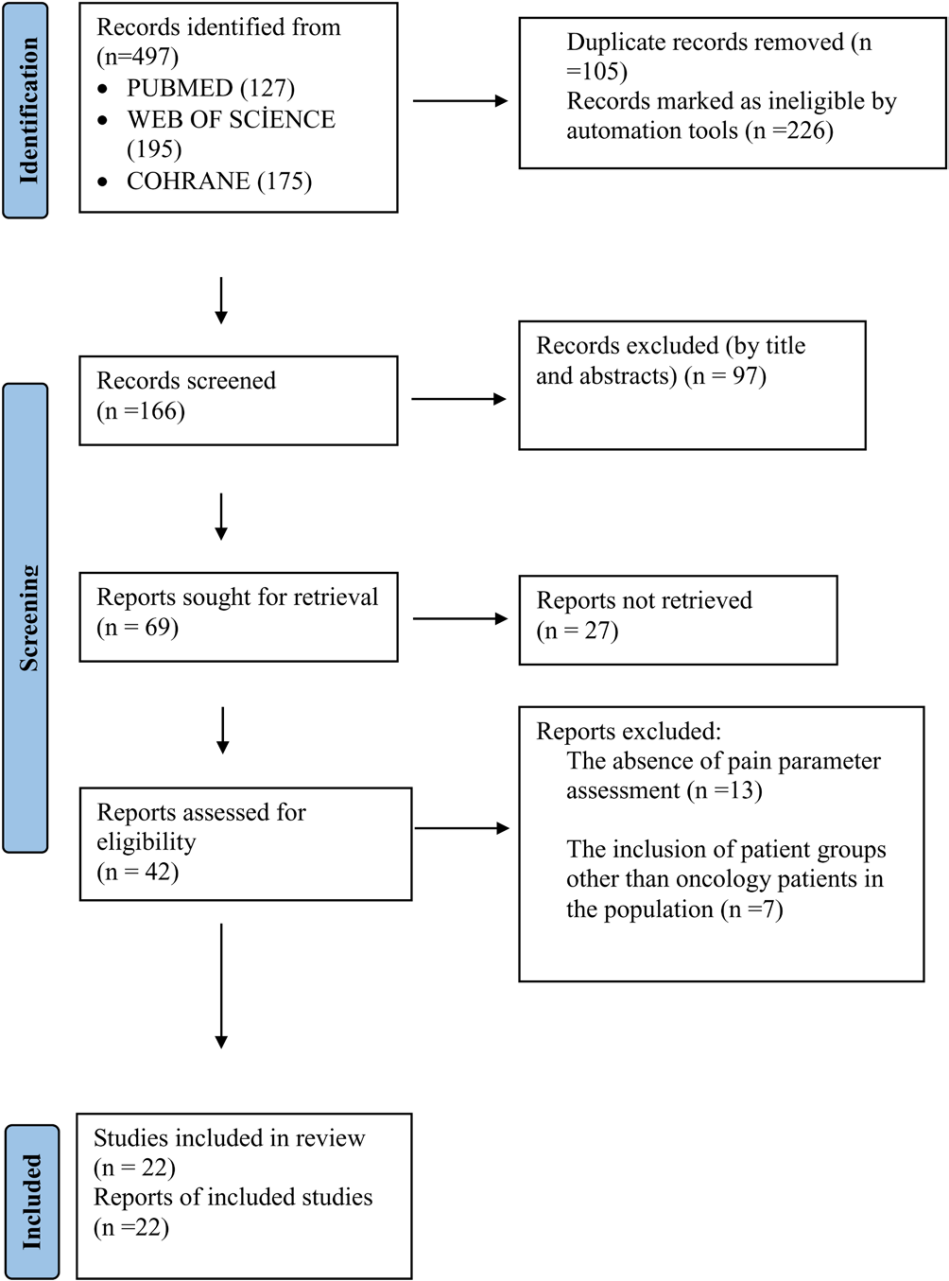
PRISMA flow diagram of the study selection process. PRISMA = Preferred Reporting Items for Systematic Reviews and Meta-Analyses.

The review was conducted through a systematic examination of experimental (ES) and quasi-experimental studies (QES) that determined the effect of VR on the pain experienced by oncology patients during their diagnosis, examination, and treatment process. The selection and review of studies were carried out via the Preferred Reporting Items for Systematic Reviews and Meta-Analyses checklist and are presented in a flow diagram (Fig. 1)^[29]^ (https://www.prisma-statement.org/prisma-2020-flow-diagram).

### 2.2. Literature search strategy

**Figure 2.**
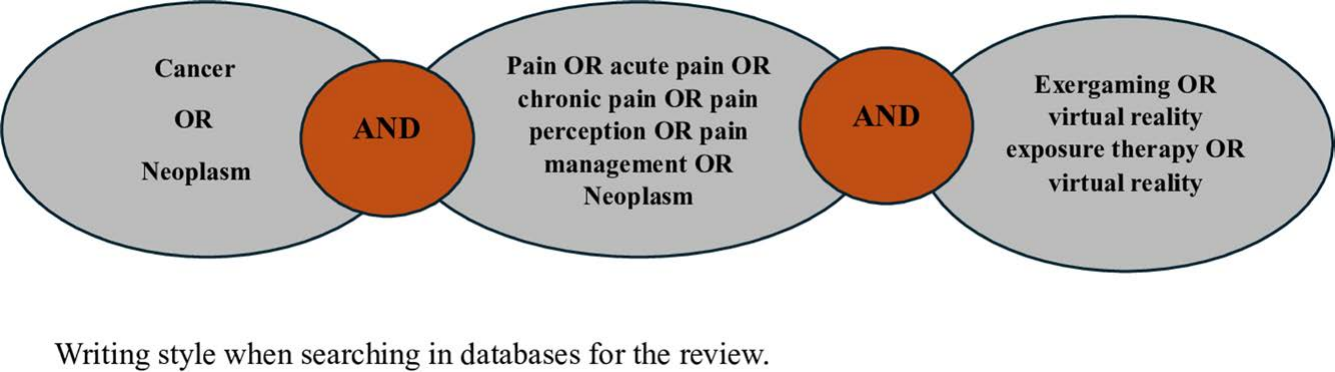
Use of terms in the search process.

The review examined studies conducted between January 1, 2014, and January 1, 2024. The literature search was performed via the PubMed, Web of Science, and Cochrane databases. The search was carried out between November and December 2024. Keywords were determined via the Medical Subject Headings index (Table 1). The search query used in the databases was “Cancer OR Neoplasm AND Pain OR acute pain OR chronic pain OR pain perception OR pain management AND Exergaming OR Virtual Reality Exposure Therapy OR Virtual Reality” (Fig. 2).

**Table 1:** Keywords in this systematic review.

MeSH term	Related keywords
Neoplasms	Cancer, Neoplasm.
Pain	Pain, Acute Pain, Chronic Pain, Pain Perception, Pain Management.
Virtual reality	Exergaming, Virtual Reality Exposure Therapy, Virtual Reality.

MeSH (Medical Subject Headings): Standardized vocabulary used for indexing and searching biomedical and health-related information.

Related keywords: Additional search terms associated with each MeSH category, used to broaden the search strategy in the systematic review.

#### 2.2.1. Inclusion and exclusion criteria

Studies that met the inclusion and exclusion criteria (Table 2) and were suitable for the PICOS (Population, Intervention, Comparison, Outcome, Study design) framework were included in the review (Table 3).

**Table 2:** Inclusion and exclusion criteria in this systematic review.

Inclusion	Exclusion
✓ The publication language must be English. ✓ The full text of the study must be accessible. ✓ The study must have been conducted in the last 10 yr (January 2014–January 2024). ✓ The study must be experimental or quasi-experimental.	✓ Being in a language other than English. ✓ Inability to access the full text of the study. ✓ Not being an experimental or quasi-experimental study. ✓ A study with a high risk of bias.

**Table 3:** PICOS Criteria in this systematic review.

P (Patient)	Individuals diagnosed with cancer
I (Intervention)	Virtual reality interventions
C (Comparison)	Standard care or non-virtual reality interventions
O (Outcome)	Pain scores after virtual reality intervention
S (Study design)	Experimental or quasi-experimental studies

PICOS is a framework used to define and structure clinical research questions:

• P (Population): The group or patients being studied.

• I (Intervention): The treatment or intervention applied.

• C (Comparison): The alternative being compared.

• O (Outcome): The desired outcome or result.

• S (Study Design): The type of study.

#### 2.2.2. Selection of studies and data extraction

The databases were selected by 2 researchers. Using search terms, 497 studies were identified. Studies found through the databases were as follows: Pubmed (127), Web of Science (195), and Cochrane (175). While using database automation tools, the following filters were applied: publication date, text availability, article type, and article language. Clinical and randomized studies published within the last 10 years, available in full text, and written in English were included. Duplicates (n = 105), records flagged as unsuitable by automation tools (n = 226), excluded on the basis of title and abstract (n = 97), articles that were inaccessible (n = 27), studies that did not assess pain as a parameter (n = 13), and studies involving populations other than oncology patients (n = 7) were excluded, leaving 22 studies included in the review. The selection process is shown in the Preferred Reporting Items for Systematic Reviews and Meta-Analyses flow diagram (Fig. 1). Data from the 22 studies were independently extracted by 2 researchers via a preprepared data collection form. The data collection form included the following information: authors, publication year, institution of the first author, title of the article, journal name, country, purpose of the article, research method, sample, sample selection method, sample size, and findings (Table 4).

**Table 4:** Characteristics of the reviewed studies.

Authors	Years of publication	Article titles	Journal title	Study purpose	Study design	Population	Sampling method	Sample size	Main findings
Gülçin Özalp Gerçeker Murat Bektaş Yeşim Aydinok Hale Ören Hülya Ellidokuz Nur Olgun	2021	The effect of virtual reality on pain, fear, and anxiety during access of a port with huber needle in pediatric hematology-oncology patients: Randomized controlled trial.	European Journal of Oncology Nursing	This study aimed to investigate the effect of VR distraction during access to the venous port with a Huber needle in reducing needle-related pain, fear, and anxiety of children and adolescents with cancer.	ES	Children and adolescents with cancer	Stratified Sampling	42 VR (21) and control (21)	Virtual reality is an effective distraction method in reducing port needle-related pain, fear, and anxiety in Pediatric Hematology-Oncology patients.
Alwin Chuan Melissa Hatty Michael Shelley Angela Lan Howard Chow E Dai Sana Haider A Bogdanovych Wei Chua	2023	Feasibility of virtual reality-delivered pain psychology therapy for cancer-related neuropathic pain: a pilot randomized controlled trial.	Anaesthesia	The aim of this study was to evaluate the feasibility, acceptability, and effectiveness of a bespoke virtual reality-delivered pain therapy software program in managing neuropathic pain in cancer patients.	ES	Cancer patients with neuropathic pain	Random Sampling	39 VR (20) and control (19)	The results showed that the virtual reality-delivered therapy was feasible, well-accepted by patients, and demonstrated potential effectiveness in reducing neuropathic pain in cancer patients.
Cho Lee Wong Chi Kong Li Carmen W H Chan Kai Chow Choi Jieling Chen Man Ting Yeung On Na Chan	2021	Virtual Reality Intervention Targeting Pain and Anxiety Among Pediatric Cancer Patients Undergoing Peripheral Intravenous Cannulation: A Randomized Controlled Trial.	Cancer Nursing	The aim of this study was to determine whether virtual reality distraction intervention can alleviate pain and anxiety and reduce length of procedure among pediatric cancer patients undergoing peripheral IV cannulation	ES	Pediatric cancer patients	Random Sampling	108 VR (54) and control (54)	Findings indicate that virtual reality is safe and effective to alleviate pain and anxiety among pediatric cancer patients undergoing PIC procedure.
Philip D Austin Philip J Siddall Melanie R Lovell	2022	Feasibility and acceptability of virtual reality for cancer pain in people receiving palliative care: a randomized cross-over study	Support Care Cancer	To determine the feasibility and preliminary effectiveness for larger randomized controlled trials of 3D head-mounted VR for managing cancer pain in adults.	ES	People receiving palliative care	Random Sampling	13 3D VR (7) and 2D VR (6)	The study has shown that both 3D virtual reality and 2D screen applications are effective in relieving pain in palliative care patients. It was stated that 3D VR offered a more immersive experience.
Remziye Semerci Melahat Akgün Kostak Tuba Eren Gülcan Avci	2021	Effects of Virtual Reality on Pain During Venous Port Access in Pediatric Oncology Patients: A Randomized Controlled Study	Pediatr Oncol Nurs	The aim of this study was to evaluate the effects of VR method on pain during venous port access in pediatric oncology patients aged 7 to 18 yr.	ES	Children with cancer (ages 7–18) undergoing port catheter access.	Random Sampling	71 VR (35) and control (36)	Virtual reality method is effective for reducing pain during venous port access in pediatric oncology patients.
Lisa M Reynolds Alana Cavadino Stanley Chin Zoë Little Amelia Akroyd Geraldine Tennant Rosie Dobson Reuben Broom Adèle Gautier	2022	The benefits and acceptability of virtual reality interventions for women with MBC in their homes; a pilot randomized trial.	MBC Cancer	The aim of this study was to assess if VR is a feasible and acceptable therapy to alleviate symptoms in women with MBC	ES	Women with MBC	Random Sampling	38 Two different VR (20 + 18)	Improvements in quality of life, fatigue, pain, depression, anxiety, and stress were seen post-intervention. Participants enjoyed the experience and were likely to use its benefits. VR had a positive effect on pain relief, though not clinically significant.
Francesco Burrai Salvatorico Ortu Marco Marinucci Maria Grazia De Marinis Michela Piredda	2023	Effectiveness of Immersive Virtual Reality in People with Cancer Undergoing Antiblastic Therapy: A Randomized Controlled Trial	Seminars In Oncology Nursing	This study aims to assess the effects of immersive VR in people with cancer undergoing antiblastic therapy, on anxiety, fatigue and pain.	ES	People with cancer undergoing antiblastic therapy	Random Sampling	74 VR (25) narrative medicine (25) standard care (24)	It was found that VR was effective in reducing anxiety and fatigue in cancer patients undergoing antiblastic treatment, but it did not change the average pain level before and after the intervention.
Amos S Hundert Kathryn A Birnie Oussama Abla Karyn Positano Celia Cassiani Sarah Lloyd Petra Hroch Tiessen Chitra Lalloo Lindsay A Jibb Jennifer Stinson	2021	A Pilot Randomized Controlled Trial of Virtual Reality Distraction to Reduce Procedural Pain During Subcutaneous Port Access in Children and Adolescents With Cancer.	The Clinical Journal of Pain.	We aimed to determine the feasibility of VR distraction for children with cancer undergoing subcutaneous port access.	ES	Children and adolescents with cancer (aged 8–18 yr) undergoing subcutaneous port needle insertion	Random Sampling	40 VR (20) and control (20)	Although not statistically significant, more VR group participants indicated no pain (65% vs 45%) and no distress (80% vs 47%) during the procedure compared with the iPad group.
Serena Moscato Vittoria Sichi Andrea Giannelli Pierpaolo Palumbo Rita Ostan Silvia Varani Raffaella Pannuti Lorenzo Chiari	2021	Virtual Reality in Home Palliative Care: Brief Report on the Effect on Cancer-Related Symptomatology	Frontiers in psychology	This study aims to assess the effect of an immersive VR-based intervention conducted at home on anxiety, depression, and pain over 4days and to evaluate the short-term effect of VR sessions on cancer-related symptomatology.	QES	Advanced cancer patients receiving home care	Purposive Sampling	14	Anxiety, depression, and pain did not change significantly between days 1 and 4. However, the Edmonton Symptom Assessment Scale items related to pain, depression, anxiety, well-being, and shortness of breath collected immediately after the VR sessions showed a significant improvement.
Özlem Feyzioğlu Selvi Dinçer Arzu Akan Zeliha Candan Algun	2020	Is Xbox 360 Kinect-based virtual reality training as effective as standard physiotherapy in patients undergoing breast cancer surgery?	Supportive Care in Cancer	This study aimed to investigate the potential effects of early postoperative VR therapy on pain, ROM, muscle strength, functionality, and fear of movement.	ES	Breast cancer who underwent unilateral mastectomy with axillary lymph node dissection and received adjuvant therapy.	Random Sampling	40 VR (20) and control (20)	Both groups detected significant changes in pain, ROM, muscle strength, grip strength, functionality, and fear of movement scores after the treatment. There were no differences in ROM, muscle strength, grip strength, and pain between the groups after the treatment.
Romain Varnier Odile Brière Thomas Brouillard Isabelle Martel-Lafay Anne-Agathe Serre Audrey Couillet Gisèle Chvetzoff Cécile Freulet Pascal Pommier	2021	Virtual reality distraction during uterovaginal brachytherapy applicators’ removal: A pilot comparative study	Brachytherapy	To assess the relevance of VR during uterovaginal brachytherapy applicators’ removal, as an alternative to nitrous oxide conscious sedation, to decrease anxiety and pain perception.	QES	Patients treated with cervical brachytherapy for locally or locally advanced cervical cancer	Convenience Sampling	35 VR (14) and control (21)	The feasibility of replacing nitrous oxide with a VR device during cervical brachytherapy was confirmed, but pain and anxiety levels were similar in both groups. The VR group did not perform better than the control group in pain management.
Özlem FEYZİOĞLU Özgül ÖZTÜRK Selvi DİNÇER Arzu AKAN	2022	Acute Effects of Video Game-based Exercises in Patients Receiving Chemotherapy After Breast Cancer Surgery - A Pilot Study.	Türk Onkoloji Dergisi	This study aimed to investigate the acute effects of a video game-based exercise program applied after breast cancer surgery on the upper extremity functionality, pain severity, and the level of fatigue.	QES	Patients who have completed 12 weeks after breast cancer surgery and are undergoing adjuvant chemotherapy treatment.	Random Sampling	15	After the video game-based exercise, no change was observed in shoulder abduction muscle strength, but an increase in pain levels was noted.
Sarah A Kelleher Hannah M Fisher Joseph G Winger Shannon N Miller Grace H Amaden Tamara J Somers Luana Colloca Hope E Uronis Francis J Keefe	2022	Virtual reality for improving pain and pain-related symptoms in patients with advanced-stage colorectal cancer: A pilot trial to test feasibility and acceptability	Palliative & supportive care	To examine the feasibility, acceptability, safety, and effectiveness of a 30-minute VR session in reducing pain and pain-related symptoms in patients with advanced colorectal cancer, and to understand preferences, thoughts, and emotions related to the VR session.	QES	Adults with advanced (stage IV) colorectal cancer.	Purposive Sampling	20	Pain decreased by 59%, tension by 74%, stress by 68%, and anxiety by 65% with VR. Positive responses were also obtained from qualitative data for the VR.
Shayesteh Sharifpour Gholam Reza Manshaee Ilnaz Sajjadian	2020	Effects of virtual reality therapy on perceived pain intensity, anxiety, catastrophising and self-efficacy among adolescents with cancer	Counselling and Psychotherapy Research	Examining the impact of VR therapy on pain outcomes in adolescent cancer patients during chemotherapy.	QES	adolescent cancer patients during chemotherapy	Convenience Sampling	30 VR (15) and control (15)	The average scores for perceived pain intensity, anxiety, catastrophizing, and self-efficacy showed greater improvement in the experimental group compared to the control group.
Sándor Erdős Klára Horváth	2023	The Impact of Virtual Reality (VR) on Psychological and Physiological Variables in Children Receiving Chemotherapy: A Pilot Cross-Over Study	Integrative cancer therapies	To investigate the effects of VR on the emotional state of pediatric oncology patients undergoing chemotherapy in a clinical setting.	QES	Pediatric oncology patients undergoing chemotherapy	Random Sampling	35 VR (21) and control (14)	The use of VR increased joy and happiness, reduced anxiety, but did not create a significant difference in other parameters. Physiological measures, such as pain, did not show changes after VR.
R Jensi Amali Seema S Chavan	2023	Effectiveness of Virtual Reality Distraction on Pain Perception and Fear among Children with Cancer Undergoing IV Cannulation	Indian journal of community medicine: official publication of Indian Association of Preventive & Social Medicine	To examine the effect of VR distraction on pain perception and fear in cancerous children undergoing IV cannulation.	QES	Cancerous children undergoing (IV) cannulation.	Convenience Sampling	80 VR (40) and control (40)	In the VR distraction group, pain perception was found to be significantly lower in both children and mothers, with a 95% confidence interval compared to the control group.
Kaylie Wilson Grace Scorsone	2021	The Use of Virtual Reality Technologies to Reduce Anxiety and Improve Experience in Chemotherapy Patients During Treatment	Frontiers in Virtual Reality	To investigate the potential psychological benefits of VR as an intervention to induce positive emotions and reduce pain levels in participants undergoing (IV) chemotherapy.	QES	INTEGRIS Kanser Enstitüsünde kemoterapi tedavisi gören hastalar	Convenience Sampling	22	Participants reported feeling calmer, more relaxed, and satisfied after using VR, but no significant changes were observed in blood pressure, pain, or anxiety levels.
Stanley Chin Alana Cavadino Amelia Akroyd Geraldine Tennant Rosie Dobson Adele Gautier Lisa Reynolds	2022	An Investigation of Virtual Reality Nature Experiences in Patients With Metastatic Breast Cancer: Secondary Analysis of a Randomized Controlled Trial	Journal of medical internet research cancer	Evaluating whether VR nature experiences provide more benefits to women with MBC who have no connection to nature compared to those who do.	QES	Women with MBC	Random Sampling	38	It was found that pain did not change over time or with connection to nature, while there was a significant reduction in fatigue, depression, and an increase in quality of life compared to baseline following the interventions.
Gregory House Grigore Burdea Namrata Grampurohit Kevin Polistico Doru Roll Frank Damiani Jasdeep Hundal Didier Demesmin	2016	A feasibility study to determine the benefits of upper extremity virtual rehabilitation therapy for coping with chronic pain post-cancer surgery	British Journal of Pain	To determine the effect of 3D games, hand grip, and positioning on pain and depression in women with pain after breast surgery.	QES	Cancer survivors with chronic upper body pain	Convenience Sampling	6	A %20 decrease trend in pain intensity was observed, confirmed by the therapist’s observations and participant feedback. Additionally, a cognitive measure showed improvement in 10 participants, and there was a significant reduction of 8.3 points in depression severity.
L Ashley Verzwyvelt Ann McNamara Xiaohui Xu Renee Stubbins	2021	Effects of virtual reality v. biophilic environments on pain and distress in oncology patients: a case-cross-over pilot study	Scientific Reports	Determining whether biophilic green therapy or a VR environment can reduce the pain and distress experienced by oncology patients during chemotherapy.	QES	Cancer patients receiving (IV) chemotherapy.	Random Sampling	33	No statistically significant differences were found between the control, Green Therapy, and VR rooms in terms of heart rate, systolic or diastolic blood pressure, salivary cortisol, pain, or distress.
Shauna Higgins Shera Feinstein Makenzie Hawkins Myles Cockburn Ashley Wysong	2019	Virtual Reality to Improve the Experience of the Mohs Patient: A Prospective Interventional Study	Journal of dermatologic surgery and oncology	To assess the benefit of VR experience in reducing anxiety or pain reported by patients after outpatient skin cancer surgery	QES	Patients who underwent Mohs micrographic surgery at the clinic.	Convenience Sampling	109	There was no statistically significant difference in patient satisfaction and pain measurements between pre- and post-survey assessments.
Jane Wong Merrylee McGuffin Mackenzie Smith Dr Andrew Loblaw	2023	The use of virtual reality hypnosis for prostate cancer patients during transperineal biopsy/gold seed implantation: A needs assessment study	Canadian journal of radiation technology	The goal was to assess patients’ interest in VR hypnosis during gold seed placement and biopsy, and to identify the subgroup of patients who would benefit the most from its use.	QES	Patients who underwent biopsy and/or gold seed placement using a two-step local anesthesia procedure.	Convenience Sampling	23	Patients with higher pain and distress scores showed more interest in trying VR hypnosis for seed placement/biopsy procedures. Those with a history of low pain tolerance or high pain levels during previous biopsies were identified as a subgroup of patients who could benefit from VR hypnosis during preradiation invasive treatment procedures.

ES = experimental studies, IV = intravenous, MBC = metastatic breast cancer, QES = quasi-experimental studies, ROM = range of motion, VR = virtual reality.

#### 2.2.3. Quality assessment

The methodological quality of the studies was evaluated to determine the extent to which potential bias was addressed from the design to the analysis of the study. The articles included in the systematic review were meticulously evaluated by 2 researchers. The assessment tools developed by the Joanna Briggs Institute (JBI) were used to evaluate the studies.^[30,31]^ These tools include questions that assess the risk of bias and whether measures related to study validity or quality are addressed. The checklist for QES contains 9 questions, and the checklist for randomized controlled trials contains 13 questions. Answers to the questions are given as “Yes,” “No,” “Unclear,” and “Not Applicable.” A “Yes” answer is given a score of 1 point, whereas other answers are given 0 points. The questions on the checklist were independently reviewed by 2 researchers. Any differing opinions were discussed, and a consensus was reached. A total of 22 studies were included for evaluation in this review: 10 ES (Table 5) and 12 QES (Table 6).

**Table 5:** Methodological quality assessment using the JBI checklist for experimental studies.

Criteria Author (yr)	1	2	3	4	5	6	7	8	9	10	11	12	13	Quality score
Gerçeker et al^[11]^	✓	?	✓	0	0	?	✓	✓	✓	✓	✓	✓	✓	9/13
Chuan et al^[12]^	✓	?	✓	0	0	?	✓	✓	✓	✓	✓	✓	✓	9/13
Wong et al^[13]^	✓	?	✓	0	0	?	✓	✓	✓	✓	✓	✓	✓	9/13
Austin et al^[14]^	✓	X	✓	0	0	X	✓	✓	✓	✓	✓	✓	✓	9/13
Semerci et al^[32]^	✓	✓	✓	0	0	X	✓	✓	✓	✓	✓	✓	✓	10/13
Reynolds et al^[21]^	✓	✓	✓	0	0	?	✓	✓	✓	✓	✓	✓	✓	10/13
Burrai et al^[19]^	✓	X	✓	0	0	?	✓	✓	✓	✓	✓	✓	✓	9/13
Hundert et al^[33]^	✓	?	✓	0	0	X	✓	✓	✓	✓	✓	✓	✓	9/13
Feyzioğlu et al^[34]^	✓	X	✓	0	0	?	✓	✓	✓	✓	✓	✓	✓	9/13
Chin et al^[35]^	✓	✓	✓	✓	0	?	?	?	✓	✓	✓	✓	✓	9/13

Symbols: ✓: Yes; X: No; ?: Unclear; 0: not applicable.

JBI = Joanna Briggs Institute.

This table presents the methodological quality assessment of included studies using the JBI Critical Appraisal Checklist for Randomized Controlled Trials, which includes the following criteria:

1. Was allocation to treatment groups concealed?

2. Were treatment groups similar at the baseline?

3. Were participants blind to treatment assignment?

4. Were those delivering the treatment blind to treatment assignment?

5. Were treatment groups treated identically other than the intervention of interest?

6. Were outcome assessors blind to treatment assignment?

7. Were outcomes measured in the same way for treatment groups?

8. Were outcomes measured in a reliable way?

9. Was follow-up complete and if not, were differences between groups in terms of their follow up adequately described and analyzed?

10. Were participants analyzed in the groups to which they were randomized?

11. Was appropriate statistical analysis used?

Was the trial design appropriate and were deviations from the standard randomized controlled trial design (individual randomization, parallel groups) accounted for in the conduct and analysis of the trial?

**Table 6:** Methodological quality assessment using the JBI checklist for quasi-experimental studies.

Criteria Author (yr)	1	2		3		4		5		6		7	8		9		Quality score
Moscato et al^[36]^	✓	X		✓		X		X		✓		✓	✓		✓		6/9
Feyzioğlu et al^[37]^	✓	X		✓		X		X		✓		✓	✓		✓		6/9
Kelleher et al^[38]^	✓	✓		✓		X		X		✓		✓	✓		✓		7/9
Sharifpour et al^[39]^	✓	✓		✓		✓		✓		✓		✓	✓		✓		9/9
Erdős and Horváth^[40]^	✓	✓		✓		✓		X		✓		✓	X		✓		7/9
Amali and Chavan^[41]^	✓	✓		✓		✓		X		✓		✓	✓		✓		8/9
Wilson and Scorsone^[16]^	✓	X		✓		✓		X		✓		✓	✓		✓		7/9
House et al^[42]^	✓	X	✓		✓		✓		✓		✓			✓		✓	8/9
Verzwyvelt et al^[43]^	✓	✓	✓		✓		✓		✓		✓			✓		✓	9/9
Higgins et al^[44]^	✓	X	✓		✓		X		✓		✓			X		✓	6/9
Wong et al^[45]^	✓	X	✓		✓		X		✓		✓			X		✓	6/9
Varnier et al^[46]^	✓	✓	✓		✓		X		✓		✓			X		✓	7/9

Symbols: ✓: Yes; X: No; ?:Unclear; 0: not applicable.

This table presents the methodological quality assessment of quasi-experimental studies included in this review, using the JBI Critical Appraisal Checklist for Quasi-Experimental Studies, which consists of the following criteria:

1. Is it clear in the study what is the “cause” and what is the “effect” are (i.e., there is no confusion about which variable comes first)?

2. Was there a control group?

3. Were participants included in any comparisons similar?

4. Were the participants included in any comparisons receiving similar treatment/care, other than the exposure or intervention of interest?

5. Were there multiple measurements of the outcome, both pre- and post-intervention/exposure?

6. Were the outcomes of participants included in any comparisons measured in the same way?

7. Were outcomes measured in a reliable way?

8. Was follow-up complete and, if not, were differences between groups in terms of their follow-up adequately described and analyzed?

9. Was appropriate statistical analysis used?

#### 2.2.4. Ethical considerations

This review is a systematic review, and no interventions were made at any institution or individual. No special approval was obtained for the review; only studies with access permission were included, and these studies are listed in the references.

## 3. Findings

This systematic review included 22 studies conducted between January 2014 and January 2024, comprising 12 QES and 10 ES (Table 4). The studies were conducted in Turkey, Australia, China, New Zealand, Italy, Canada, France, the United Kingdom, Iran, Hungary, the United States, and India.

### 3.1. Methodological quality assessment

The 10 ES were evaluated according to the “JBI Checklist for Experimental Studies.” These studies scored a minimum of 9 and a maximum of 10 points out of 13, with an average score of 9.2 (Table 5). The QES were assessed via the “JBI Checklist for Quasi-Experimental Studies.” These studies scored between 6 and 9 points out of 9, with an average score of 7.17 (Table 6).

The results of this review are presented under 3 main headings: the characteristics of the cancer patients addressed in the review, the VR applications used in the review, and the pain assessment scales and results used in the review.

### 3.2. Characteristics of the cancer patients addressed in the review

As a result of the studies examined in the review, a total of 925 participants were reached. The age range of the sample varies from 6 to 85 years. Among the participants, 503 were women (54.38%), and 352 were men (38.05%). The gender of 70 participants (7.57%) was not specified.^[39,41]^ Some studies have focused on specific sex groups related to cancer diagnosis, such as breast, prostate, and cervical cancers.^[20,21,35,37,42,45,46]^ The included studies also examined specific types of cancers, such as cervical, colorectal, skin, and prostate cancers.^[21,34,35,37,38,42,44–46]^ Additionally, some studies involve heterogeneous patient groups with various cancer diagnoses. These heterogeneous groups include hematologic cancers, such as leukemia and lymphoma^[14,16,33,36,40,41,45]^ gastrointestinal cancers such as pancreatic, stomach, and esophageal cancers^[14,16,19,36]^ and various other types such as osteosarcoma, laryngeal, testicular, brain, ovarian, endometrial, bone and soft tissue, endocrine system, and urinary system cancers.^[16,19,33,39,40]^

### 3.3. Virtual reality applications used in the review

The duration of VR applications varies from 3 minutes to 50 minutes. However, Wong et al^[45]^ did not specify the duration of VR use in their study. In some studies, multiple VR applications were used and participants were allowed to choose different hardware and content were used in the studies.^[11,16,19,41]^

The hardware used includes Samsung Gear Oculus,^[11,39,40]^ Oculus Rift S^[12,14]^ Google Cardboard glasses,^[13]^ Pico Goblin headset^[21,35]^ Oculus Quest 2,^[19,43]^ unnamed headsets,^[16,33,36,38,41,45,46]^ Piranha™ VR,^[32]^ Mirage Solo VR,^[36]^ Xbox Kinect™,^[34,37]^ and the BrightArm Duo Rehabilitation.

In terms of content, it appears that VR technologies aim to allow users to engage in physical or mental interaction through themes such as nature, sports, games, art, and entertainment. Nature experiences were generally the most preferred method. The participants were provided with experiences such as a sense of navigation, exploration, and task completion. Different environments, such as beaches, mountains, rivers, lakes, waterfalls, islands, and deserts, are preferred.^[14,16,19,21,35,36,40,42–44]^

Another preferred area was underwater experiences. These include coral reefs, shipwrecks, and swimming with underwater creatures.^[11,33,36,38,39,44,46]^

Additionally, amusement park rides, sports and skill-focused games, treasure hunts, and animation viewing are among the various entertainment, exercise, and gaming experiences included in these studies.^[11–13,32,34,36,37,41,42,44]^

### 3.4. Pain assessment scales and results used in the review

Various tools have been used to measure pain in studies. Among the most frequently used tools are the visual analog scale^[13,19,33,34,37,38,40,45,46]^ and the modified brief pain inventory (BPI).^[12,21,35,36,38]^ These scales include the Numerical Rating Scale ^[14,42–44]^ and the Wong-Baker FACES Pain Rating Scale.^[11,32,41]^ Additionally, the studies used the European Organization for Research and Treatment of Cancer Quality of Life Questionnaire–Core 30, the Neuropathic Symptom and Sign Scale,^[12]^ the Edmonton Symptom Assessment Scale,^[14,36]^ the McGill Pain Questionnaire,^[39]^ the Likert scale,^[16]^ the Pain Anxiety Symptoms Scale, the Pain Catastrophizing Scale, the Pain Self-Efficacy Questionnaire, and the Children’s Fear Scale (CFS).

In addition to these scales, parameters such as the use of opioids,^[12]^ physiological effects (e.g., heart rate, blood pressure, and electrodermal activity), and psychological effects (e.g., happiness, joy, fear, anxiety, irritability, alertness, patience, and nausea) have also been used to assess pain.^[40]^ Sharifpour et al^[39]^ used the Pain Anxiety Symptoms Scale, Pain Catastrophizing Scale, and Pain Self-Efficacy Scale in their study.

The majority of studies included in the review revealed that VR interventions were effective in pain management.^[11–14,21,32,33,36,38,39,41,42,45]^ However, there are also studies that report no significant change in pain due to VR intervention^[16,19,35,40,43,44]^ In one of those studies, VR interventions were reported to have a negative impact on pain.^[37]^ There are also studies that suggest that VR applications and rehabilitation programs are effective for pain but are not superior to standard treatments.^[34,46]^

## 4. Discussion

Oncology patients experience different types and intensities of pain during diagnosis, treatment, and rehabilitation processes. Technological advancements offer new and effective approaches to manage these pains, with VR applications emerging as one of the leading methods in this field. Given its increasing use, evaluating the impact of VR on pain perception in oncology patients is considered an important step in enhancing the quality of patient care.^[19,39]^ This review evaluated the effects of VR on pain experienced by oncology patients from various age groups and clinical backgrounds. It is anticipated that these findings will significantly contribute to clinical practice and the literature.

The studies discussed in this review involve patient groups with various cancer diagnoses. Among the cancer types examined are breast, cervical, colorectal, skin, prostate, hematologic, neuroendocrine system, gastrointestinal system, and urogenital system cancers^[14,16,19,21,33–42,44–46]^ The sample groups in these studies were determined on the basis of common characteristics such as age, type of treatment, procedures applied, and cancer stage. Patient groups include children and adolescents with cancer,^[11,13]^ patients receiving port or intravenous catheter treatments^[33,39,41]^ patients with advanced-stage cancer, patients with neuropathic pain, patients receiving palliative care,^[12,14,36]^ and individuals receiving chemotherapy and antineoplastic treatments.^[16,19,39,40,43]^ In the literature, the use of VR applications in a wide range of patient groups is also evident. The impact of VR on pain management has been evaluated in middle-aged and elderly cancer patients,^[24]^ individuals with chronic musculoskeletal pain,^[47]^ and patients recovering from heart surgery.^[48]^ Additionally, the analgesic effects of VR during procedures that cause high levels of pain, such as childbirth and burn dressing changes, as well as during minimally invasive surgical interventions, have been studied.^[49]^ These findings demonstrate the expanding clinical application areas of VR and its effectiveness in different patient groups.

In the studies included in this review, VR is presented through various themes, such as entertainment, exercise, experiencing nature, being in different environments, and exploration. The VR applications in the literature also display similar content. For example, applications involving active video games played with music,^[50]^ activity-based training,^[51]^ and experiencing nature with auditory and visual stimuli^[52]^ are examples of such VR-based content. Lee et al used VR for educational purposes, making the process more engaging by presenting medical procedures to children through animated characters. In conclusion, although the designs are different, the VR content reviewed here is also widely used in the literature.^[53]^

In most of the studies included in the review, VR applications have been found to be effective in pain management.^[11–14,21,32,33,36,38,39,41,42,45]^ These results are consistent with those of the study by Grassini, which examined the effectiveness of VR applications in chronic pain.^[54]^ Similarly, O’Connor et al reported that VR applications were beneficial for pain management but emphasized the need for larger and more robust studies due to the limitations of existing research.^[55]^ In their review, Chen et al^[24]^ highlighted the importance of VR-based therapies for pain management in middle-aged and elderly cancer patients.

Some studies within the reviewed research suggest that VR interventions do not result in significant changes in pain.^[16,19,35,40,43,44]^ Burrai et al^[19]^ explained this by stating that 75% of the participants did not experience pain before and after the intervention. In the literature, a similar result was reported in the study by Micheluzzi et al^[48]^, who reported that, compared with standard care, VR did not result in statistically significant differences in pain among heart surgery patients.

Some studies indicate that VR applications and rehabilitation programs are effective in pain management but are not superior to standard treatments.^[34,46]^ This could be explained by the use of effective interventions, such as analgesics and physiotherapy, in the control group.

In only one study, VR application was found to have a negative impact on pain. Feyzioğlu et al^[37]^ reported that video game-based exercises led to an increase in pain intensity in the postoperative period. This increase was attributed to the active exercises performed for 30 minutes, which moved the upper extremities in all directions, and the fact that these exercises were performed on days when the patients were not receiving chemotherapy.

Some studies in the review examined participants’ experiences and satisfaction with VR applications. Overall, the applications had positive effects on the groups. Participants described their VR experiences as pleasurable, peaceful, enjoyable, providing an escape, relaxing, soothing, exciting, stimulating exploration, attention-grabbing, motivating, and evoking childhood memories.^[11,13,14,16,19,21,34–37,39,40,42–44]^ In some studies, even though VR had no significant impact on pain, VR applications generated positive emotions in participants.^[16,35,40,43,44]^ Wong et al^[45]^ also reported that patients with high pain levels were more willing to try VR. This finding indicates that individuals are more willing to use increasing amounts of technological applications. Zanatta et al reported that despite different emotional responses to VR, the acceptability of VR was high.^[56]^

Although satisfaction with VR applications has been reported in most studies, some studies have highlighted mild and tolerable side effects. These side effects include nausea, dizziness^[12,14,21,36,38,46]^ temporary feelings of claustrophobia,^[21]^ mild eye fatigue and headaches,^[19]^ vomiting,^[46]^ and pain and fatigue.^[37]^ Some patients reported feeling more pain at the beginning of the treatment, but this pain decreased as the treatment progressed.^[42]^ In addition, discomfort and hesitation related to wearing headsets were observed.^[43,44]^

## 5. Conclusion and recommendations

This review revealed that the use of VR applications is increasing, with a variety of options and content being offered. It appears that VR applications are generally effective in pain management. Even in studies where there was no significant impact on pain, the overall satisfaction levels of participants were found to be high. To make VR more effective in pain management, it is important to conduct higher-quality studies and increase sample sizes. Furthermore, adapting VR content on the basis of the characteristics of the sample group or giving participants the option to choose content may be beneficial. Considering the effects of VR technologies on pain management in cancer patients, it is critical for healthcare professionals to be more knowledgeable about this innovative treatment method. Increasing research on the integration of VR applications into clinical settings is also crucial. This review is expected to contribute to future clinical studies.

## 6. Limitations and strengths

This study provides a comprehensive review systematically evaluating the effects of VR technology on pain management in cancer patients over the last decade. Findings from a large study covering various types of cancer and different patient groups have made it possible to consider the benefits of VR applications in various contexts. The methodological quality of the included studies was meticulously evaluated by 2 researchers and analyzed according to the JBI criteria, which enhances the reliability of the results. Moreover, this review not only focused on pain but also examined several important factors, such as patient satisfaction, psychological changes, and side effects as discussed in the articles.

A limitation of this study is that only studies published in English and with full-text access were included. The methodological differences between the included studies may limit the generalizability of the results. For example, variations in the duration of VR applications, types of content, and hardware used have created heterogeneity in the results. Additionally, some studies had small sample sizes, which limits the statistical power of the results. Furthermore, the lack of control over variables such as participant age, cancer type, and treatment stage may reduce the generalizability of the findings.

## 7. Implications for nursing practice

The results of this review demonstrate the potential of VR applications in pain management and psychological relief for cancer patients. Oncology nurses can provide more effective and holistic care to their patients by utilizing VR technology as a tool for pain management. Therefore, it is crucial for nurses to receive training on the operation, effects, and usage methods of VR applications. Training programs should cover not only the use of VR but also appropriate patient selection, the preference for individualized content, and the management of potential side effects.

When VR applications are integrated into existing care plans, nurses should collect patient feedback regularly and assess their levels of satisfaction. Additionally, VR applications can be combined with other non-pharmacological methods to develop a more holistic pain management strategy. Oncology nurses can use this technology not only for pain management but also for alleviating psychological symptoms such as anxiety, fear, and stress. This will help provide a more positive treatment experience for both patients and their families. Finally, oncology nurses can plan new studies to strengthen evidence on the effectiveness of VR applications in patients with different cancer diagnoses to promote the widespread use of VR in clinical settings.

## Acknowledgments

We would like to thank all the nurses who contributed to this research.

## Author contributions

**Conceptualization:** Ayşin Tepe, Elif Dönmez.

**Formal analysis:** Ayşin Tepe, Elif Dönmez.

**Methodology:** Ayşin Tepe, Elif Dönmez.

**Project administration:** Ayşin Tepe, Elif Dönmez, Tülay Ortabağ.

**Resources:** Tülay Ortabağ.

**Visualization:** Ayşin Tepe.

**Writing – original draft:** Ayşin Tepe.

**Writing – review & editing:** Ayşin Tepe, Tülay Ortabağ, Elif Dönmez.
